# Mesenchymal cell differentiation during lymph node organogenesis

**DOI:** 10.3389/fimmu.2012.00381

**Published:** 2012-12-14

**Authors:** Andrea Brendolan, Jorge H. Caamaño

**Affiliations:** ^1^Division of Molecular Oncology, San Raffaele Scientific InstituteMilan, Italy; ^2^School of Immunity and Infection, Institute for BioMedical Research-Medical Research Council Centre for Immune Regulation, College of Medical and Dental Sciences, University of BirminghamBirmingham, UK

**Keywords:** lymphoid tissues, stromal cells, lympho-mesenchymal interactions, lymphotoxin beta receptor, NF-κB

## Abstract

Secondary lymphoid tissues such as lymph nodes are essential for the interactions between antigen presenting cells and lymphocytes that result in adaptive immune responses that protect the host against invading pathogens. The specialized architecture of these organs facilitates the cognate interactions between antigen-loaded dendritic cells and lymphocytes expressing their specific receptor as well as B–T cell interactions that are at the core of long lasting adaptive immune responses. Lymph nodes develop during embryogenesis as a result of a series of cross-talk interactions between a hematopoietically derived cell lineage called lymphoid tissue inducer cells and stromal cells of mesenchymal origin to form the anlagen of these organs. This review will present an overview of the different signaling pathways and maturation steps that mesenchymal cells undergo during the process of lymph node formation such as cell specification, priming, and maturation to become lymphoid tissue stromal organizer cells.

Lymph nodes (LN) develop during embryogenesis in mice and humans following a precise timing depending on anatomical location. Mesenteric LNs develop first in mouse embryos around embryonic day (E) 10.5, followed by the rest of these organs along the anterior-posterior body axis (Rennert et al., 1996, 1998; Mebius, 2003). Vertebrate organogenesis results from complex interactions of molecular and cellular networks in which progenitor cells become specified, proliferate and differentiate to ascertain organ formation and function (Brendolan et al., 2007; Costantini and Kopan, 2010). These sequential events are orchestrated by signaling molecules that activate cell-specific gene expression programs in uncommitted progenitors (Dressler, 2009). Thus, it is conceivable that LN development relies on similar mechanisms for the acquisition of cellular identity (specification) and that uncommitted mesenchymal cells assume a LN fate prior to proliferation and formation of the anlagen. Once specified, mesenchymal cells engage in cross-talk with lymphoid cells and this assures LN expansion coupled to mesenchymal cell differentiation (Roozendaal and Mebius, 2011). Two central cellular players required for the development of secondary lymphoid tissues during mouse embryogenesis have been identified (Honda et al., 2001; Mebius, 2003; Nishikawa et al., 2003). Lymphoid tissue inducer (LTi) cells, derived from lymphoid cell precursors and belonging to the family of innate lymphoid cells and mesenchymal progenitors cells whose origin have not been elucidated yet. LTi cells express CD45, CD4, interleukin-7 receptor α, integrin α4β7, receptor activator of NF-κB (RANK/TRANCE-R) and its ligand RANKL/TRANCE, lymphotoxin α1β2 (LT1β2), and the chemokine receptor CXCR5 and thus are attracted in response to the chemokine CXC-chemokine ligand 13 (CXCL13) secreted by mesenchymal cells.

Conversely, mesenchymal cells have been characterized as CD45^–^, PDGF-receptor α^+^, lymphotoxin β receptor^+^ (LTβR), vascular cell adhesion molecule-1 (VCAM-1^–^), and intercellular adhesion molecule-1 (ICAM-1^–^). Analysis of different knockout mouse models has led to the discovery of several genes required for LN development and revealed a multistep process in which interactions between LTi cells and mesenchymal cells appear crucial to assure organ formation. However, the origin and identity of the signals that induce the specification of mesenchymal progenitor cells prior to the arrival of LTi cells at the site of LN formation remain largely unknown.

Lymphoid tissue inducer cell numbers appear to be the limiting factor controlling the development of LNs and other secondary lymphoid tissues as shown by the fact that over-expression of IL-7 *in vivo*, results in a significant increase of LTi cells and the number of LN (Meier et al., 2007).

## MESENCHYMAL CELL SPECIFICATION

The mechanisms governing the spatial and temporal organization of the different LNs are poorly understood. It is currently unclear which signals assure LN organogenesis to take place at define locations along the body axis and ensure mesenchymal cell specification prior to the clustering of LTi cells at sites where these organs will develop.

**FIGURE 1:**
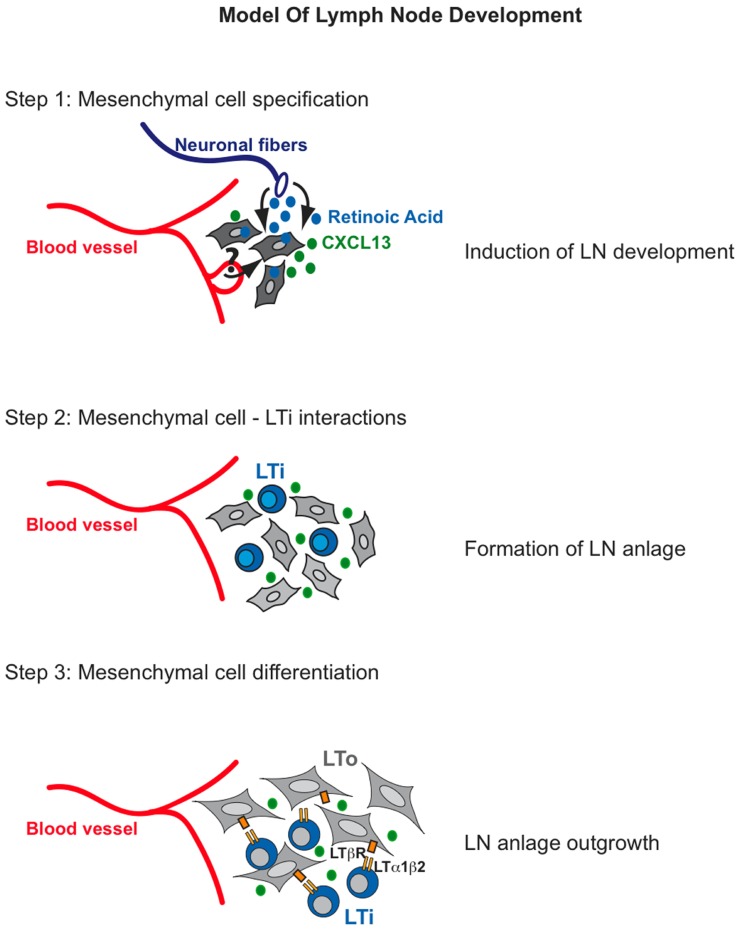
**Model of lymph node development**. *Step 1*: Retinoic Acid produced by neurons stimulates mesenchymal cells to express the chemokine CXCL13. *Step 2*: CXCL13 expression by mesenchymal cells attracts lymphoid tissue inducer (LTi) cells to the site where lymph nodes will develop. LTi cells will cluster and they might signal in trans to each other through RANKL-RANK. *Step 3*: RANK signaling on LTi cells will induce high expression levels of LTα1β2. Binding of the latter to LTβR on mesenchymal cells will induce the expression of cell adhesion molecules VCAM-1, ICAM-1, and MAdCAM-1 as well as CXCL13, CCL21, and CCL19 to initiate a positive feedback loop that will attract large numbers of LTi cells to the LN anlage and thus result in the formation of the structure the organs.

Given the different location of LNs along the antero-posterior axis, it is likely that different signals are required to commit progenitor cells toward a LN fate for each specific set of organs. Expression of CXCL13 by mesenchymal cells appears to be the first sign of mesenchymal cell specification (van de Pavert et al., 2009; **Figure 1**, step 1). This chemokine is required for the initial recruitment of LTi cells to form clusters with the former at the sites of LN formation (Ansel et al., 2000; Luther et al., 2003; Ohl et al., 2003). Recent work has shown that retinoic acid (RA) from neuronal cells induces CXCL13 expression in mesenchymal cells, indicating a possible role for RA in specifying, at least in part, the LN-mesenchyme (van de Pavert et al., 2009). These results suggest a mechanism for specification of mesenchymal cells and for their initial condensation to form the LN anlagen that is LTi cell-independent. While RA was shown to induce CXCL13 expression in the mesenchymal LN anlage, it is presently unknown whether this morphogen has a similar action in the presumptive mesenchymal cells at the site of all LNs or different specification signals exist depending on the location of the organs. *Cxcl13*^–/–^ and *Cxcr5*^–/–^ embryos fail to form most LN anlagen due to the inability of recruiting LTi cells, yet mesenteric and cervical LNs are present in adult *Cxcl13*^–/–^ mice, and *Cxcr5*^–/–^ embryos develop rudimentary LN anlage (Ansel et al., 2000; Ohl et al., 2003). However, the finding that specification of mesenchymal cells can occur even in the absence of CXCL13 argues in favor of other signals required for this process.

Given the pleiotropic role of RA during vertebrate development including its capacity to regulate cell-fate and differentiation (Niederreither and Dolle, 2008), if RA derived from neuronal cells is required for specifying the mesenchymal cells of most LN, it is likely that, in addition to CXCL13, it activates a set of downstream targets including Hox genes (Wang et al., 2006; Duester, 2008). Hox genes encode a family of homeodomain transcription factors required for organ patterning and cell fate specification along the antero-posterior axis during embryogenesis thus they represent candidate factors involved in patterning the early LN anlage. It has been shown that the mature counterpart of the mesenchymal cells that form the LN anlage, lymphoid tissue organizer (LTo) cells from mesenteric LNs and Peyer’s patches express different Hox genes (Okuda et al., 2007).

A century ago, Sabin (1902, 1909) identified a series of structures in pig embryos named lymph sacs from which it was hypothesized that LN develop. In support of this hypothesis is the recent finding that lymph sacs form during inguinal LN formation through endothelial-cell budding from the vasculature. It was proposed that invasion of the endothelial lumen by the surrounding mesenchyme will form the LN anlagen (Benezech et al., 2010). However, Vondenhoff et al. (2009b) have shown that in mouse embryos lacking lymphatic vasculature due to conditional ablation of *Prox1* in endothelial cells (*Tie2-Cre*;* Prox1*^*f*/*f*^) the LN anlage still forms, although it is associated with hypoplasia and reduced number of LTi cells. While these findings indicate that lymphatic endothelial cells and lymph sacs may not be required for positioning LTi cells at the site of organ formation, they do not exclude a role for the lymphatic endothelium in specifying the surrounding mesenchyme. Thus, loss of lymphatic signals may affect the ability of mesenchymal cells to become fully specified and to attract a critical number of LTi cells at the site of LN anlage formation and this scenario may also explain the LN hypoplasia observed in *Prox1*^–/–^ mice.

At present, however, despite a possible role of RA in the specification of mesenchymal progenitors, the source of the signals and the transcriptional program that ensures full commitment of progenitor cells into LN mesenchymal cells remains unknown.

## LYMPH NODE ANLAGE FORMATION AND EXPANSION

Following mesenchymal cell specification and clustering of LTi cells, a series of cross-talk interactions between these cell populations appear to be crucial to assure organ development (**Figure 1**, step 2). Clustering of LTi cells expressing both RANK-L and its receptor RANK in the developing LN anlagen suggest that the RANK-L/RANK signaling pathway might be activated in trans by the close proximity of these cells (Kim et al., 2000; Vondenhoff et al., 2009a). RANK signaling up-regulates the expression of LTα1β2 thus enhancing the cross-talk interactions between LTi cells and the mesenchymal precursor cells (Yoshida et al., 2002; Vondenhoff et al., 2009a). A putative function for RANK signaling in LN stromal cells has been recently indicated (Hess et al., 2012; Sugiyama et al., 2012). Analysis of the CD45^–^ cell population in mesenteric and inguinal LN of E14 mouse embryos onward shows the presence of newly specified mesenchymal cells that are negative for VCAM-1 and ICAM-1 expression but positive for PDGFRα. By E15.5 these V^–^I^–^ mesenchymal precursors differentiate into VCAM-1^Int^ ICAM-1^Int^ PDGFRα^+^ “primed” mesenchymal cells independently of the presence of LTi cells as accumulation of this intermediate population is present in *Rorc*^–/–^ and *Ltβr*^–/–^ embryonic LNs (Benezech et al., 2010). Importantly, the signal/s that prime the V^–^ I^–^ progenitors to become V^int^ I^int^ mesenchymal cells remains to be identified. By E17.5 primed V^int^ I^int^ mesenchymal cells differentiate into V^high^ I^high^ MAdCAM-1^+^ LTo cells (**Figure 1**, step 3). This maturation step requires LT signaling and LTi cells as LTo cells are absent in the LN anlagen of *Ltbr*^–/–^ and *Rorc*^–/–^ mice (lacking LTi cells). Accumulation of LTi cells in the LN anlagen coincides with the differentiation of V^int^ I^int^ mesenchymal cells into V^high^I^high^ MAdCAM-1^+^ LTo cells (Cupedo et al., 2004b; Cupedo and Mebius, 2005; Benezech et al., 2010).

The tumor necrosis factor (TNF) family ligand LTα1β2 expressed on LTi cells engages its receptor, LTβR on V^int^ I^int^ mesenchymal cells, resulting in the activation of the NF-κB family of transcription factors through the classical/canonical (NF-κB1 p50/RelA) and the alternative/non-canonical pathways (NF-κB2 p52/RelB; Dejardin et al., 2002; Yilmaz et al., 2003). This process leads to increased expression of ICAM-1 and VCAM-1 and the homeostatic chemokines CXCL13, CCL19, and CCL21 creating a positive feedback loop for the continuous recruitment and retention of LTi cells and for the proliferation and homeostasis of LTo cells (Randall et al., 2008; Ruddle and Akirav, 2009; van de Pavert and Mebius, 2010). Interestingly, while the three stromal cell populations present in LN anlagen (V^–^I^–^, V^int^ I^int^, V^high^I^high^) express LTβR, the NF-κB member RelB is only detected in V^int^ I^int^, and V^high^I^high^ cells correlating with their higher levels of expression of chemokines and cell adhesion molecules with respect to the V^–^I^–^ cell population (Benezech et al., 2010). Lack of LTβR signaling in LTo cells, as observed in *Ltα*^–/–^, *Ltβr*^–/–^, and *Rorc*^–/–^ mice, results in the absence of all LNs (De Togni et al., 1994; Futterer et al., 1998; Sun et al., 2000; Eberl and Littman, 2003; Eberl et al., 2004; White et al., 2007; Benezech et al., 2010). *Relb*^–/–^ mice also fail to develop all LNs and *Nfkb2*^–/–^ mice present with poorly developed inguinal and popliteal LNs due to impaired expression of chemokines, cell adhesion molecules and development of high endothelial venules (Weih and Caamaño, 2003; Carragher et al., 2004). Similarly, mice carrying a phosphorylation mutant kinase IKKα, that is essential for the activation of the NF-κB alternative pathway, have a similar LN phenotype than the *Nfkb*2^–/–^ mice (Drayton et al., 2004). LTβR signaling is required for the maturation and homeostasis of LTo cells and for expression of RANK-L, MAdCAM-1, and lympho-organogenic chemokines (Yoshida et al., 2002; Eberl et al., 2004; Coles et al., 2006; White et al., 2007; Vondenhoff et al., 2009a; Benezech et al., 2010).

## LN ORGAN EXPANSION AND LT ORGANIZER CELL DIFFERENTIATION

Lymphoid tissue organizer cells are thought to represent the precursors of mature stromal cells in adult LN. However, the contribution of the former to the stromal cell subsets in adult organs and the signals that induce their differentiation are still poorly defined.

Previous work showed that transplantation of neonatal LN cells under the skin of adult mice gave rise to distinct stromal cell networks, thus indicating that neonatal LTo cells from the transplanted cell suspensions were capable to differentiate into mature stromal cells (Cupedo et al., 2004a). Importantly depletion of LTi cells from the neonatal LN cell populations impaired their ability to develop an ectopic lymphoid structure in this system. Despite that these findings indicate that neonatal LN contains stromal progenitors, lineage-tracing experiments are required to unequivocally demonstrate the precursor-product relationship between embryonic LTo cells and adult LN stromal cells, including follicular dendritic cells as previously suggested (Katakai et al., 2008). In addition, it also needs to be determined whether the different stromal cell subsets originate from single multipotent mesenchymal progenitors or whether distinct progenitors exist for each stromal cell subpopulation.

## MARGINAL RETICULAR CELLS

Lymphoid tissue organizer cells and marginal reticular cells (MRCs) are similar in the expression of several markers suggesting a precursor-product relationship between these cell types (Katakai et al., 2008; Roozendaal and Mebius, 2011), although formal proof that LTo cells can generate MRCs is still lacking. MRCs are located under the subcapsular sinus of LNs and in the spleen marginal zone where they are referred as marginal sinus lining cells. These stromal cells express MAdCAM-1, CXCL13, VCAM-1, ICAM-1, BP3, and RANK-L and their maturation does not appear to depend on signals from T- or B-cells as *Rag*2^–/–^ mice have an intact LN MRC layer (Katakai et al., 2008). However, blocking LTβR signaling causes loss of CXCL13 and MAdCAM-1 expression on MRCs thus indicating that engagement of the LT pathway, possibly by LTi cells or their adult counterpart is required for maintaining the phenotypic characteristics of these cells. A recent report has shown that over-expression of RANK-L has an effect on MRCs and other stromal and endothelial cell types in adult LN resulting in enhance cell proliferation and organ expansion with increase numbers of B-cell follicles (Hess et al., 2012). Conversely, blocking of RANK-L in mouse embryos appears to disrupt B-cell follicle formation and induce HEV maturation in newborn mice (Sugiyama et al., 2012). In addition, differentiation of MRC appears to be strictly connected to signals associated to the development of secondary lymphoid organs since these cells are not found in ectopic lymphoid tissues and tertiary lymphoid organs (Katakai et al., 2008).

## FOLLICULAR DENDRITIC CELLS

Follicular dendritic cells (FDCs) localize in the center of the B-cell follicle and appear a week after birth. Several studies have proposed a mesenchymal origin for FDCs although it is currently unclear whether FDCs arise from in situ embryonic mesenchymal precursors or from cells migrating to the organ postnatally (Allen and Cyster, 2008; e.g., bone marrow).

Recent studies show that FDCs originate from perivascular progenitor cells expressing *Mfge8 *and *Pdgfrb* genes and that ablation of PDGFRβ^+^ cells induces the collapse of FDC networks. In addition, these findings also showed that PDGFRβ^+^ perivascular cells from non-lymphoid organs have the capacity to differentiate into FDCs *in vitro* and *in vivo*, thus suggesting that this cell population may be the source of FDC in tertiary lymphoid organ formation (Krautler et al., 2012). B-cell derived signals are required for FDC maturation as demonstrated by mice deficient for TNFα, LTα_1_β_2_ and their receptors that fail to develop FDC networks and germinal centers (Allen and Cyster, 2008).

## FIBROBLASTIC RETICULAR CELLS

Fibroblastic reticular cells (FRCs) are a heterogeneous population of stromal cells distributed in the T-zone of secondary lymphoid organs (Mueller and Germain, 2009). FRCs form the conduit system, a network of collagen-rich channels surrounded by fibroblasts that allows small molecules, such as chemokines and antigens to reach the T cell zones (Sixt et al., 2005; Bajenoff et al., 2006). Contrary to the spleen in which the formation of the FRC network depends on LTα1β2 from B-cells, LNs FRC networks develop normally in the absence of B-cells (Ngo et al., 2001), thus indicating that different signaling molecules and cell types may be required for proper FRC differentiation in different lymphoid organs. At present, it remains unclear whether FRCs originate from a common embryonic mesenchymal progenitor or if different lineages of mesenchymal cells generate the FRC network.

## CONCLUDING REMARKS

Over the past several years, novel findings have highlighted the complexity of the cellular and molecular mechanisms governing lymphoid organ development and function. Central to these findings is the notion that interactions between lymphoid and mesenchymal cells are crucial for the development of secondary lymphoid organs. However, the cellular and molecular events underlying LN regionalization and those implicated in mesenchymal cell specification remain largely undefined.

It is also unknown at what point during lineage diversification mesenchymal cells become fully committed toward a specific fate and whether distinct stromal cell subsets arise from single multipotent progenitors or if different precursors exists for each stromal cell type. Despite these developmentally unsolved questions, recent work by several groups has shown that stromal cells are not merely passive inhabitants of lymphoid organs as previously thought, but instead are active players in modulating the activity of the immune system by providing structural support and signals for survival, attraction, locomotion, and activation of immune cells (Mueller and Germain, 2009). The recent discovery that some stromal cell subsets contribute to tolerance induction further highlights their important function in the homeostasis of immune system (Fletcher et al., 2011). Thus, a full understanding of the ontogeny and function of the stromal microenvironment still requires that we uncover the genetic and transcriptional programs underlying mesenchymal cell differentiation and elucidate the molecular repertoire that characterize each stromal subsets during normal and pathological conditions.

## Conflict of Interest Statement

The authors declare that the research was conducted in the absence of any commercial or financial relationships that could be construed as a potential conflict of interest.
